# Pre-pandemic Predictors of Loneliness in Adult Men During COVID-19

**DOI:** 10.3389/fpsyt.2021.775588

**Published:** 2021-12-08

**Authors:** Kayla A. Mansour, Christopher J. Greenwood, Ebony J. Biden, Lauren M. Francis, Craig A. Olsson, Jacqui A. Macdonald

**Affiliations:** ^1^Centre for Social and Early Emotional Development, School of Psychology, Faculty of Health, Deakin University, Geelong, VIC, Australia; ^2^Centre for Adolescent Health, Murdoch Children's Research Institute, Royal Children's Hospital, Melbourne, VIC, Australia; ^3^Department of Paediatrics, University of Melbourne, Royal Children's Hospital, Melbourne, VIC, Australia

**Keywords:** male, COVID-19, loneliness, longitudinal, pandemic

## Abstract

Loneliness is a major public health issue, with its prevalence rising during COVID-19 pandemic lockdowns and mandated “social distancing” practices. A 2020 global study (*n* = 46,054) found that, in comparison to women, men experienced the greatest levels of loneliness. Although research on predictors of loneliness during COVID-19 is increasing, little is known about the characteristics of men who may be particularly vulnerable. Studies using prospective data are needed to inform preventative measures to support men at risk of loneliness. The current study draws on rare longitudinal data from an Australian cohort of men in young to mid-adulthood (*n* = 283; aged *M* = 34.6, SD = 1.38 years) to examine 25 pre-pandemic psychosocial predictors of loneliness during COVID-19 social restrictions (March–September 2020). Adjusted linear regressions identified 22 pre-pandemic predictors of loneliness across a range of trait-based, relational, career/home and mental health variables. Given the extensive set of predictors, we then conducted penalized regression models (LASSO), a machine learning approach, allowing us to identify the best fitting multivariable set of predictors of loneliness during the pandemic. In these models, men's sense of pre-pandemic environmental mastery emerged as the strongest predictor of loneliness. Depression, neuroticism and social support also remained key predictors of pandemic loneliness (*R*^2^ = 26, including covariates). Our findings suggest that men's loneliness can be detected prospectively and under varying levels of social restriction, presenting possible targets for prevention efforts for those most vulnerable.

## Introduction

Loneliness is a preventable public health issue and has been linked to mental illness, suicide, poor health behaviors, and premature death (1, 2). It is characterized by a perceived lack of social support and a sense of social disconnection (3, 4) and is often stigmatized or trivialized (5). Emerging evidence indicates loneliness is increasingly prevalent, with a global survey (*n* = 23,004) finding 1 in 3 adults experience feelings of loneliness (6). Concerns about loneliness have escalated in the context of COVID-19 pandemic lockdowns and mandated “social distancing” practices, with loneliness rising since the first recorded SARS-CoV-2 infections and representing one of the strongest predictors of depression, anxiety, and post-traumatic stress disorder during the pandemic (7–9). Much of the research to date on loneliness during the pandemic has focused on adolescents (10), or older adults (11–15) or has been limited to investigations of demographic factors (16). Here we extend this work to identify psychosocial factors among men in their young- to mid-adult years who maybe particularly vulnerable (17, 18).

A 2020 global study capturing data from 237 countries, islands, and territories (*n* = 46,054) found that, in comparison to women, men experienced the greatest levels of loneliness, particularly young men in “individualistic” societies (e.g., America or Australia) (17). Where individualistic values such as self-reliance and personal autonomy intersect with gendered expectations of men, there may be reduced access and use of social support (19), which may increase risk for loneliness (20). An Australian national survey found men aged 25–44 years who lived alone experienced higher rates of loneliness (39%) than women living alone (12%) (18). This disparity may reflect higher levels of emotional distancing in men, compared to women (21, 22). Relatedly, a 2019 multi-national survey (*n* = 4,000) found that almost 50% of adult men felt they could not or would not talk to friends about their problems (23). Furthermore, young- to mid-adulthood is the normative age for consolidating relationships and becoming a father (24), yet even during this period of life, almost one in four new fathers report feeling isolated (23).

Rates of loneliness in adult men are particularly alarming when considering meta-analytic evidence that shows over one-third of the variance in suicidal ideation and behavior among men is explained by loneliness, although these findings were not age specific (25). Concerns are compounded by the exceedingly high male suicide rates globally (26). In Australia, where this study was conducted, male suicide is three times greater than the rate for females, and in young adult men up to 34 years of age, suicide accounts for 32.6% of all deaths (27). Loneliness has also been associated with higher rates of mental health problems both before and during the pandemic (4, 28). Cross-sectional pandemic research has found that loneliness during the pandemic is associated with higher rates of mental health problems across both genders (8, 29, 30). In a Polish study of adults aged 18–35 years (*n* = 380), loneliness was associated with symptoms of mental health problems and increased concern about COVID-19's health threat (30). In pre-pandemic research, loneliness similarly predicted heightened stress appraisals (31) and threat perception (32), suggesting that individuals with high levels of loneliness may assess the pandemic in a more threatening way and therefore be at greater risk of mental health problems or distress (30).

Given the elevated risk of loneliness for all individuals during the pandemic, the high prevalence of loneliness in adult men pre-pandemic (17), and the potential mental health consequences of loneliness (2), further understanding loneliness in men under pandemic conditions is warranted. In particular, an understanding of psychosocial predictors of loneliness in men during the pandemic under varying levels of social restrictions would provide information on who is most vulnerable and the degree to which lockdown and related restrictions are relevant to this relationship. Factors associated with loneliness, identified in prior research, fall largely within trait-based, relational, career/home and mental health domains (33, 34). At the trait level, loneliness has been associated with the “Big Five” personality traits (35), particularly (low) extraversion and (high) neuroticism (36), as well as constructs such as (low) self-efficacy (37). Relational factors linked to loneliness include lack of social activity and reduced quality of relationships with peers, family, and significant others (38). Across the career and home domain, aspects such as skills and satisfactions have been associated with loneliness (39, 40). In the mental health domain, loneliness has been associated with greater levels of depression, anxiety, and generalized distress (41). However, across all domains, most research has been cross-sectional, analyses often do not report effects of gender, or the predictors or correlates are usually selected from within a single domain (33, 42). Research is yet to prospectively assess a complex set of predictors for adult male loneliness that may help to identify the best set of variables for detecting future vulnerability, particularly during times of social restriction.

The COVID-19 pandemic has been a precipitating or exacerbating event for loneliness and may therefore reveal new insights into factors predicting vulnerability to a sense of social disconnection experienced by many men. The current study draws on rare longitudinal data from an Australian cohort of men in young to mid-adulthood. Our aims were three-fold. First, we sought to separately examine prospective associations between a suite of pre-pandemic variables across the multiple domains and loneliness assessed at two time points across the first year of the COVID-19 pandemic (2020). Second, we aimed to identify if associations differed depending on varying levels of COVID-19 social restrictions. Third, we sought to determine the relative contribution of predictors on the levels of men's loneliness. To achieve the final aim, we used the Least Absolute Shrinkage and Selection Operator (LASSO) penalized regression (43), a machine learning approach, allowing us to identify the best fitting multivariable set of predictors of loneliness during the pandemic.

## Materials and Methods

### Participants

Participants were from the Men and Parenting Pathways (MAPP) Study (*N* = 608), a longitudinal cohort study that examines the mental health and wellbeing of Australian men across the peak age for transitioning to fatherhood (33 years) (44). Men aged between 28 and 32 years (inclusive) were recruited between 2015 to 2017 from all states and territories of Australia via social media, partnerships with community and private organizations, as well as word of mouth. Three annual waves of data collection, with a participation rate of 83% across waves 2 or 3, were complete prior to the first cases of COVID-19 being detected in the world (45).

In March 2020, the Australian federal, state, and territory governments announced a national response to the COVID-19 pandemic that included the shutdown of non-essential industries and the directive to “stay home” except for four reasons: (1) shopping for essential items, (2) care and caregiving, (3) exercise, and (4) essential study or work—if unable to do so from home (46). In the same month, a 15-min survey (open between March 21st to May 19th) was added to the MAPP study to capture the impact of the COVID-19 pandemic on the lives of MAPP cohort participants. The stay-at-home restrictions led to a decrease in cases across the country; however, by June there was a rapid spike in COVID-19 cases in the State of Victoria, where 42% of this sample of participants reside. As a result, the Victorian State Government enforced one of the world's strictest lockdowns at the time, where an 8 p.m. curfew and a directive to stay within a 5 km radius from home was enforced, in addition to the prior set of restrictions. During this period (July 20th to September 2nd) MAPP participants who participated in the first COVID-19 specific survey were invited to complete a second COVID-19 survey.

To be included in the current study, participants were required to have provided data on one or both of the MAPP COVID-19 surveys and be living in Australia at the time of the survey. The analytic sample were 283 adult men aged between 32 to 38 years at the time of the second COVID-19 survey (*M* = 34.6, *SD* = 1.38). In comparison to the original MAPP sample, the analytic sample showed no differences on key baseline characteristics including socio-economic advantage and disadvantage (SEIFA), employment, birthplace, ethnicity, parenting status, and sexuality, however, they were more highly educated. The original MAPP sample has been compared against the general Australian population of men at this age, see the MAPP Cohort Profile paper for more information (44).

### Measures

#### Outcome Measure

At both COVID-19 timepoints, loneliness was measured with the 8-item University of California, Los Angeles Loneliness Scale (ULS-8) (47) to examine the level of social contact experienced compared to what is desired. The scale includes statements such as “*I lack companionship*” scored on a 4-point Likert scale where 1 = *Never*, 2 = *Rarely*, 3 = *Sometimes*, and 4 = *Always*. The total score ranges from 8 to 32 points. Higher scores reflect greater levels of perceived loneliness. Scores ≥ 25 indicate very high levels of loneliness with chronic experience of at least one symptom (i.e., “always” endorsed in response options). Scores between 17 and 24 inclusive indicate moderate to high levels of periodic loneliness with an average endorsement of “sometimes” as a response option. The ULS-8 has a Cronbach's alpha of 0.83 and has been found to be a valid substitute for the full 20-item version of this scale, the ULCA-20 (47). For the current study, participants' maximum level of loneliness reported across our two COVID-19 waves was used.

#### Predictor Measures

Twenty-five predictors across individual, relational, and mental health domains were included in analyses to measure risk factors of loneliness. Data for each variable were taken from participants' most recent pre-COVID-19 response across waves 1–3. If data were missing from wave 3, information from wave 2 was given preference, followed by wave 1. Information on the predictor variables and their associated scales are presented in Table 1.

**Table 1:** Pre-pandemic predictor measures.

**Construct**	**Scale**	**No. items**	**Response options**	**Possible score range**	**Reliability (Cronbach's Alpha)**
**Trait-based**					
Openness to experience	Mini IPIP-6	4	1 = Very to 7 = Very accurate	4–28	0.71
Conscientiousness	Mini IPIP-6	4	1 = Very to 7 = Very accurate	4–28	0.63
Extraversion	Mini IPIP-6	4	1 = Very to 7 = Very accurate	4–28	0.81
Agreeableness	Mini IPIP-6	4	1 = Very to 7 = Very accurate	4–28	0.77
Neuroticism	Mini IPIP-6	4	1 = Very to 7 = Very accurate	4–28	0.69
Honesty-humility	Mini IPIP-6	4	1 = Very to 7 = Very accurate	4–28	0.70
Trait anger	STAXI-2	10	1 = Almost to 4 = Almost always	10–40	0.88
Socially prescribed perfectionism	MPS	15	1 = Disagree to 7 = Agree	15–105	0.87
**Relational**					
Hours spent with friends	Single item	1	Open ended	Per week	-
Social support	MSPSS	12	1 = Very Strongly Disagree to 7 = Very Strongly Agree	12–84	0.92
History of maternal care	PBI	12	1 = Very like to 4 = Very unlike	0–36	0.92
History of maternal control	PBI	13	1 = Very like to 4 = Very unlike	0–39	0.88
History of paternal care	PBI	12	1 = Very like to 4 = Very unlike	0–36	0.93
History of paternal control	PBI	13	1 = Very like to 4 = Very unlike	0–39	0.87
**Career and home orientation**					
Career orientated identity salience	ISS	5	1 = Strongly disagree to 5 = Strongly agree	4–20	0.79
Job competence	BPNQ	14	1 = Not at all true to 5 = Very true	14–70	0.54
Home competence	BPNQ	14	1 = Not at all true to 5 = Very true	14–70	0.54
**Mental health and wellbeing**					
Depression	DASS-21	7	0 = Did not apply to me at all to 3 = Applied to me very much, or most of the time	0–42	0.92
Anxiety	DASS-21	7	0 = Did not apply to me at all to 3 = Applied to me very much, or most of the time	0–42	0.86
Stress	DASS-21	7	0 = Did not apply to me at all to 3 = Applied to me very much, or most of the time	0–42	0.89
Environmental mastery	PWB	7	1 = Strongly Disagree to 6 = Strongly Agree	7–42	0.84
Purpose in life	PWB	7	1 = Strongly Disagree to 6 = Strongly Agree	7–42	0.83
Poor overall physical health	Single item	1	1 = Excellent to 5 = Poor	1–5	-
State anger	STAXI-2	15	1 = Not at to 4 = Very much so	15–60	0.95
Irritability	BITe	5	1 = Never to 5 = Always	5–25	0.91

*Mini IPIP-6, Mini International Personality Item Pool Six-Items (48); STAXI-2, State-Trait Anger Expression Inventory 2 (49); MPS, Multidimensional Perfectionism Scale (50); Hours spent with friends, How many hours a week on average, outside of work, would you spend in the company of friends? MSPSS, Multidimensional Scale of Perceived Social Support (51); PBI, Parental Bonding Instrument (52); ISS, Identity Salience Scale (53); BPNQ, Basic Psychological Needs Questionnaire (54); DASS-21, Depression Anxiety Stress Scale- 21 Items (55); PWB, Ryff's Scales of Psychological Wellbeing (56); Overall Physical Health, How would you rate your physical health (poor to excellent)?; BITe, The Brief Irritability Test (57)*.

#### Covariates

We sought to examine the predictive nature of psychosocial factors on men's loneliness net of baseline and contextual factors. Therefore, in adjusted regression analyses, we included baseline demographic covariates that had previously been linked to increased levels of loneliness (16, 58, 59). These were income (0 = >$AUD 60,000 per annum, 1 = ≤ $AUD 60,000 per annum), birthplace (0 = Australia, 1 = not Australia), and education (0 = >high school education, 1 = ≤ high school completion). We also included relevant contextual factors measured at the time of the pandemic to investigate if the associations were net of varying pandemic experiences. Contextual covariates included living alone (0 = not living alone, 1 = living alone), time spent online socializing with friends (0 = 3 or more times per week, 1 = <3 times per week), and current state of residence (0 = non-Victoria, 1 = Victoria) given the extended lockdown period Victoria experienced in comparison to the rest of Australia as described earlier.

### Statistical Analysis

Data were cleaned and derived in Stata 15 (60). Analyses were conducted in R version 4.0 statistical software (61). First, linear regression analyses were used to examine associations between participants' maximum levels of loneliness during the pandemic and each pre-pandemic predictor. Analyses were estimated unadjusted and then repeated adjusting for all covariates. To address our second aim, interactions were tested to examine whether state of residence (Non-Victoria vs. Victoria) during the pandemic moderated the relationship between loneliness and each predictor variable. To address the third aim, LASSO models, a type of penalized regression, were then estimated to develop a predictive model and identify key indicators of loneliness during the pandemic. In comparison to traditional methods, LASSO shrinks coefficient sizes by applying a penalty factor and retains important predictor variables (i.e., coefficients greater than zero) (62, 63). This is advantageous over traditional regression models as it reduces overfitting and in turn improves predictive performance in new data (62). Further, this method produces simpler and more interpretable models with a reduced set of the predictors (63). A more detailed description of the LASSO model is available elsewhere (43, 62). To tune the strength of the penalty factor, 5-fold cross-validation was used, whereby the training data is split into 5 equal datasets, referred to as folds. Models with a range of penalty strengths are iteratively trained using 4-folds and then tested on the remaining fold. This process is repeated five times, such that all folds are used for testing (62). Penalty strength is selected based on the predictive performance across testing folds. Predictive performance of the LASSO was assessed across 100 iterations of training and testing data splits 80/20% (62) via *R*2 in the testing split. For each iteration the LASSO identified: (1) the “best” model, which minimizes predictor error out of the sample, and (2) the “one-standard-error” model, where the out-of-sample prediction error is within one standard error of the “best” model, resulting in a more parsimonious solution (62). To determine the most robust predictors, LASSO models were re-run using the full sample and 100 iterations of 5-fold cross-validation. The mean of the coefficients was obtained for predictors that were selected in at least 80% of the cross-validation iterations (62). A single imputed data set was generated using the R *mice* package (64) due to standard penalized regression packages in R not having built in capacity to handle multiple imputed or missing data. All continuous variables were standardized (z-scored) prior to analyses. A series of traditional linear regressions were conducted post LASSO model selection to confirm the relative contribution of covariates and key predictors identified. The covariates were regressed onto the outcome variable of loneliness and the coefficient of determination (*R*^2^) was examined. The key predictors identified in each of the LASSO models were added to the regression and the *R*^2^ was again examined.

## Results

### Descriptives

Six percent of the analytic sample reported very high levels of loneliness during the pandemic, while a majority (56%) reported moderate to high levels. Table 2 presents a summary of the outcome, pre-pandemic predictors, and covariates. Pairwise correlations between predictors can be found in Supplementary Table 1.

**Table 2:** Descriptive statistics of the outcome, pre-pandemic predictors, and covariates.

	**M**	**SD**	**Missing (%)**
**Outcome**			
Loneliness (maximum) during COVID-19	18.08	4.29	0%
**Pre-pandemic predictors**			
Trait-based			
Openness to experience	20.91	4.49	8%
Conscientiousness	17.68	4.21	8%
Extraversion	15.29	5.00	8%
Agreeableness	19.87	4.70	8%
Neuroticism	14.52	4.67	8%
Honesty-Humility	19.65	4.94	8%
Trait anger	18.16	5.81	8%
Socially prescribed perfectionism	55.18	13.21	10%
Relational			
Hours spent with friends	5.24	6.99	1%
Social support	62.80	13.67	1%
History of maternal care	26.30	7.21	8%
History of maternal control	13.31	7.21	8%
History of paternal care	20.80	8.69	10%
History of paternal control	10.64	7.04	10%
Career and home orientation			
Career orientated identity salience	12.41	3.96	2%
Job competence	22.66	4.05	0%
Home competence	24.80	4.84	1%
Mental health and wellbeing			
Depression	10.61	10.22	0%
Anxiety	6.42	7.40	1%
Stress	12.96	9.25	1%
Environmental mastery	27.60	6.25	0%
Purpose in life	29.07	6.41	2%
Poor overall physical health	3.05	1.04	2%
State anger	29.64	9.75	1%
Irritability	14.15	4.10	1%
**Covariates**	**n**	**%**	**Missing (%)**
Education (low)	36	13%	0%
Income (low)	30	10%	0%
Birthplace (Australia)	252	88%	0%
State of residence (Victoria)	119	42%	0%
Living alone (yes)	22	8%	0%
Online socializing with friends (<3 times a week)	203	71%	0%

### Predictors of Loneliness Across COVID-19

#### Traditional Regression Models

Table 3 shows prospective associations between pre-pandemic predictors and participant reported loneliness during the pandemic. Unadjusted analyses and analyses adjusting for all covariates provided evidence for an association between each of the predictors and pandemic loneliness with the exception of agreeableness, honesty-humility, and openness to experience. Specifically, at the trait-based level, neuroticism, trait anger, and perfectionism were positively associated with loneliness during the pandemic (β range = 0.19–0.43), while extraversion, conscientiousness, were negatively associated (β range = −0.15 to −0.26). At the relational level, hours spent with friends, social support, and retrospective accounts of parental care were negatively associated with pandemic loneliness (β range = −0.16 to −0.40), while retrospective accounts of parental control were positively associated (β range = 0.15–0.16). At the career and home orientated level, job competence and home competence were negatively associated with pandemic loneliness (β range = −0.05 to −0.34), while career orientated identity salience was positively associated (β = 0.12). At the mental health and wellbeing level, positive associations were found with depression, anxiety, stress, state anger, irritability, and poor overall physical health (β range = 0.26–0.48) and negative associations with environmental mastery and purpose in life (β range = −0.08 to −0.33). The pattern of associations was similar in the unadjusted models. Additionally, for all predictor variables, no evidence emerged for an interaction with state of residence (Supplementary Table 2).

**Table 3:** Associations between pre-pandemic predictors and subsequent pandemic loneliness.

**Variable**		**Unadjusted**				Adjusted^*^				
	**β**	**95%**	**CIs**	***p***	**β**	**95%**	**CIs**	***p***	Penalized regression β (Best fit)^*^	Penalized regression β (1 SE)^*^
**Trait-based**										
Openness to experience	−0.02	−0.13	0.10	0.801	−0.03	−0.14	0.09	0.639		
Conscientiousness	−0.18	−0.30	−0.07	0.002	−0.15	−0.27	−0.035	0.011		
Extraversion	−0.26	−0.37	−0.15	<0.001	−0.26	−0.37	−0.147	<0.001	−0.04	
Agreeableness	−0.05	−0.17	0.07	0.407	−0.02	−0.14	0.091	0.680	0.04	
Neuroticism	0.44	0.33	0.55	<0.001	0.43	0.32	0.54	0.000	0.13	0.07
Honesty-humility	−0.04	−0.15	0.08	0.501	−0.02	−0.14	0.09	0.682		
Trait Anger	0.22	0.11	0.33	<0.001	0.19	0.08	0.31	0.001		
Socially prescribed perfectionism	0.32	0.21	0.43	<0.001	0.32	0.21	0.43	<0.001		
**Relational**										
Hours spent with friends	−0.15	−0.26	−0.03	0.014	−0.16	−0.27	−0.05	0.005		
Social support	−0.45	−0.55	−0.34	<0.001	−0.40	−0.51	−0.30	<0.001	−0.08	−0.03
History of maternal care	−0.16	−0.27	−0.04	0.008	−0.16	−0.27	−0.04	0.007		
History of maternal control	0.17	0.05	0.28	0.004	0.15	0.04	0.27	0.006		
History of paternal care	−0.20	−0.31	−0.09	0.001	−0.19	−0.30	−0.08	0.001		
History of paternal control	0.20	0.08	0.31	0.001	0.16	0.05	0.27	0.006		
**Career and home orientation**										
Career orientated identity salience	0.16	0.04	0.27	0.008	0.12	0.00	0.232	0.040		
Job competence	−0.06	−0.08	−0.03	<0.001	−0.05	−0.08	−0.02	<0.001		
Home competence	−0.33	−0.44	−0.22	<0.001	−0.34	−0.45	−0.23	<0.001	−0.02	
**Mental health and wellbeing**										
Depression	0.50	0.40	0.60	<0.001	0.48	0.37	0.584	<0.001	0.16	0.11
Anxiety	0.28	0.17	0.40	<0.001	0.26	0.15	0.372	<0.001		
Stress	0.33	0.22	0.44	<0.001	0.31	0.20	0.415	<0.001		
Environmental mastery	−0.08	−0.10	−0.07	<0.001	−0.08	−0.10	−0.07	<0.001	−0.21	−0.20
Purpose in life	−0.37	−0.48	−0.26	<0.001	−0.33	−0.45	−0.22	<0.001		
Poor overall physical health	0.30	0.18	0.41	<0.001	0.29	0.19	0.40	<0.001	0.04	
State anger	0.31	0.20	0.42	<0.001	0.27	0.16	0.383	<0.001		
Irritability	0.36	0.25	0.47	<0.001	0.34	0.23	0.448	<0.001		
									*R*^2^ = 0.30	*R*^2^ = 0.26

* *Adjusted for education, income, birthplace, state of residence, living alone, and online socializing with friends*.

#### LASSO Penalized Regression Models

The more parsimonious LASSO model (i.e., the one-standard-error model) selected environmental mastery as the strongest pre-pandemic predictor for loneliness during the pandemic (β = −0.20), followed by depression (β = 0.11), neuroticism (β = 0.07), and social support (β = −0.03). Together, this LASSO model (including covariates) explained 26% of variance in loneliness. In the best fitting model, the same pre-pandemic predictors were selected and had the strongest associations, with the addition of a further four variables, including extraversion (β = −0.04), agreeableness (β = 0.04), overall physical health (β = 0.04), and home competence (β = −0.02), accounting for an additional 4% of the variance in loneliness. To identify the relative contribution of covariates and key predictors selected by the LASSO models, we conducted subsequent traditional regression analyses that showed the six covariates accounted for 9.5% of the variation in loneliness [*F*_(6, 276)_ = 4.86, *p* < 0.001]. When the four key predictors from the “one-standard-error” model were added to the model the amount of variance explained increased more than four-fold to 39.5% [*F*_(10, 272)_ = 17.75, *p* < 0.001]. The additional four predictors from the “best-fit” model accounted for a further 2.2% of variance in loneliness [*F*_(14, 268)_ = 13.69, *p* < 0.001].

## Discussion

This study presents findings from a unique Australian longitudinal study of men with the aim of addressing a key gap in knowledge about pre-pandemic predictors of loneliness under socially restrictive, pandemic conditions. In our sample, 6% of men reported high levels of loneliness, endorsing “always” feeling at least one symptom of loneliness such as lacking companionship. A further 56% of men indicated moderate to high levels of loneliness during the pandemic and associated lockdown periods. With respect to aim one, we found evidence of associations between loneliness and 22 of the 25 pre-pandemic predictors we examined before and after adjustment for covariates. In the relational, career/home and mental health and wellbeing domains, all variables predicted loneliness. In the trait domain, only five of the eight traits assessed predicted loneliness. Regarding aim two, all associations were not influenced by living in the state of Victoria where an extended stay at home order was enforced, suggesting associations with loneliness may be relevant across varied experiences of lockdown restrictions. For aim three, the strongest and most consistently selected predictors of loneliness, identified in penalized regression models, were pre-pandemic low environmental mastery, depressive symptoms, neuroticism, and low perceived social support. Additional predictors identified were low extraversion, agreeableness, poor overall physical health, and a low sense of competence in completing home life tasks. The identified predictors accounted for a substantial amount of variance, net of covariates. Our findings bring to light indicators of men's subjective experience of loneliness that existed for men who experienced both short and extended periods of lockdown and were evident regardless of levels of online social interaction and other covariates.

Environmental mastery was the strongest independent pre-pandemic predictor of loneliness, suggesting that higher pre-pandemic mastery in men may confer protection against loneliness during the pandemic. Environmental mastery is characterized by one's ability to manage stressful events and a sense of control over the external world (65, 66). Although some situational factors relevant to mastery such as predictability and choice (67) were lessened in the context of government mandates on pandemic behaviors, associations remained. Our findings align with a recent meta-analysis (*k* = 6, *n* = 3,827) that reported an aggregated negative association (*r* = −0.33) between environmental mastery and loneliness (68). One explanation for this may be that individuals with high levels of mastery are more resilient to stressful life events (68), and employ more active coping strategies, such as approaching others for support, which may mitigate loneliness (69, 70). Further, in line with cognitive models of stress management, those with low mastery may appraise restrictions and the uncontrollable “loss” of connection to others more acutely (65). With past research also demonstrating a direct relationship between mastery and social support (71), men with high mastery may have been more likely to approach others for support during the pandemic, therefore protecting against loneliness. There is some evidence of higher average levels of environmental mastery in men compared to women (72), suggesting that this may be a particularly important psychological asset for men under conditions of stress.

Consistent with a sizeable body of research, we found pre-pandemic depressive symptoms to be the next strongest predictor men's pandemic loneliness (73–75). Symptoms of depression such as anhedonia, low energy, and hopelessness, can be taxing on interpersonal relationships, particularly when symptoms are chronic or episodes are frequent (76). This can lead to abandonment of social connections and social isolation (73, 74). In longitudinal studies, depressive symptoms have been associated with a reduction in the formation and maintenance of social ties, as well as a termination of previous ones (73, 74). The bi-directionality of the relationship between depressive symptoms and loneliness (75) may also result in persistent symptoms of both. Given high rates of men reporting low levels of social connections (77), understanding the potential perpetuating cycle of depression and loneliness remains key for future research.

We found neuroticism to also predict pandemic loneliness in men. Neuroticism is characterized by negative affect and increased levels of distress (78), which may underlie the inherent emotional content of loneliness that accompanies a sense of disconnection. That is, neuroticism may partially explain feelings of distress when it is perceived that there is no-one who can or will meet one's own social needs (31, 79). Additionally, individuals high on trait neuroticism tend to perceive the world as threatening and find it difficult to manage stressful life events (80). Prior COVID-19 pandemic research reported a positive association between neurotic traits and accepting and employing social distancing guidelines to avoid infection (81, 82). In this respect, individuals with high neuroticism may have heightened levels of concerns around pandemic consequences and related information (79), and therefore engage more stringently in self-isolation practices during COVID-19 that heighten risk of loneliness.

The final of the four key predictors of loneliness in our LASSO models was social support. Our findings align with a vast body of pre-pandemic research that has identified social support provided by friends, family, and significant others, as a protective factor associated with reduced levels of loneliness (41, 83). An earlier COVID-19 pandemic study found that individuals with high levels of perceived social support during the pandemic were 89% less likely than those with low levels of social support to be classed with the highest levels of loneliness (84). We extended on this research demonstrating that men's social support prior to the pandemic predicted their loneliness during the pandemic. Social support engenders a sense of camaraderie, comfort, and healthy interdependence on one another for support within an individual's social network especially during times of increased need. This may assist in maintaining supportive connections into the future, even in the context of physical distancing (41, 85).

The link between social support and loneliness may be particularly relevant to public health initiatives targeting men given that on average, men tend to report lower levels of perceived social support than women (86–88). Masculine ideals that promote stoicism, and stigma attached to seeking support may reduce some men's likelihood of having adequate social support available during times of heightened need (89). Additionally, when experiencing feelings of loneliness, sadness, and distress, some men express difficulty or feelings of embarrassment disclosing this information to a loved one and therefore reduce the likelihood of eliciting support (77). Our research suggests that even in the context of multiple established risk factors, men's low perceived social support stands out as a key indicator of future risk of loneliness.

The four additional predictors of loneliness unique to the best fit penalized regression model were pre-pandemic poor overall physical health, low extraversion, agreeableness, and low home competence (additional *R*^2^ = 0.04). Selection of extraversion and agreeableness, in addition to neuroticism, suggests that personality traits are important predictors of loneliness. Lower levels of extraversion (introversion) predicted loneliness, which is consistent with past research (36). Extraversion has been linked to the formation of social ties online, and therefore during periods of restriction, extraverts may have adapted more easily to online strategies for communication with others within their support network during the pandemic thus reducing feelings of loneliness (90). While, agreeableness was a weak predictor of loneliness, these findings should be interpreted with caution given that there was an inverse direction of this association at the bivariate level with wide confidence intervals. In saying this, LASSO prediction models are not guided by *p*-values (43, 62). Therefore, even though agreeableness was *p* < 0.05 in the traditional model, it still improved the predictive performance of the outcome, in combination with the other predictors. Loneliness during the pandemic was also predicted by poorer health pre-pandemic. Poor health has been shown to have an indirect influence on loneliness through social participation and social resources (91). Home competence was also predictive of loneliness and may be specifically relevant for pandemic related loneliness when a “stay at home” directive was enforced and a sense of efficacy in managing tasks may have promoted positive mood and wellbeing and reduced attention on negative outcomes.

Loneliness is a major public health concern for men in young to mid adulthood (17). We presented rare, community-based, longitudinal data on men in young to mid adulthood; however, evidence indicates that the developmental origins of loneliness are likely to be prior to adulthood (92). Boys, up to the early adolescent years, typically openly express their desire for genuine social connections, particularly with other boys, however, as they reach middle to late adolescence, there can be a “crisis of connection,” characterized by a loss of close intimate friendships with other males (93). Longitudinal, qualitative research shows this can perpetuate into early and mid-adulthood (94). With a growing body evidence suggesting considerable vulnerability for loneliness in men in early to mid-adulthood (18), we provide critical information on who might be at greatest risk of feeling disconnected. This is particularly important given that at this stage of life, men are at the normative age for becoming fathers and their feelings of emotional connection or disconnection may have ramifications for family wellbeing (23).

### Strengths and Limitations

There are a number of strengths and limitations in our study that should be noted. As is common in longitudinal studies, MAPP experienced some loss to follow-up, however, retention of participants has been high in comparison to other cohort studies of men (44, 95). The use of self-report measures for all predictors and the outcome measure of loneliness also poses as a possible limitation of this research with concerns around response bias, including social desirability (96). Despite this, the ULS-8 loneliness scale has been shown to be a consistent and valid measure, and the mean levels of loneliness reported by the men in our study were in line with a Norwegian study (*N* = 10,061) when strict social distancing measures had been implemented (97). It is also important to note the low reliability of the two competence scales. Further, most measures were examined prospectively, however, the Parental Bonding Instrument (PBI) is a retrospective assessment of prior relationships in childhood and adolescence. While recall bias may be present, two 20-year longitudinal studies found the measure to be stable and resistant to mood state (98, 99). We used LASSO models because they improve predictive performance when multiple factors are under consideration and increase the likelihood of replication in other samples (62). Further, the breadth of risk and protective predictors included in our analyses allowed for a wide examination across a variety of trait-based, relational, career/home and mental health and wellbeing domains. These should be understood alongside previously identified demographic factors such as education, income, and living arrangements (16). However, there are other possible relevant predictive factors that we did not include such as self-efficacy beliefs, cognitive functioning and feelings of safety in the community, which have been previously linked to feelings of loneliness (100).

### Conclusions

Our study presents novel findings within a community-based sample of early to mid-adult men to address a key gap in knowledge about predictors of loneliness, specifically under restrictive pandemic conditions. More than half of the men in our sample indicated at least periodic moderate to high levels of loneliness, and 22 of 25 pre-pandemic risk and protective factors we examined across individual, relational, and mental health and wellbeing domains predicted loneliness. Our analytic technique allowed us to refine this to a set of the four most robust predictors of men's loneliness. These were environmental mastery, depressive symptoms, neuroticism, and low perceived social support. Using rare, longitudinal data, this study has implications for programs seeking to target men who may be vulnerable to feeling lonely and experiencing its associated risks. These factors may be used in healthcare settings to aid in screening for risk of loneliness. The prospective identification of risk for loneliness represents a vital opportunity for preventing the distressing effects of perceived social disconnection experienced by men. Further, predictors identified in this model warrant investigation in studies that can assess whether they are causally related to loneliness which may inform intervention development.

## Data Availability Statement

The data analyzed in this study is subject to the following licenses/restrictions: As per our data sharing policy, MAPP ethics approvals do not include participant consent for public availability of our data, however, requests for reuse of data for validation, verification or confirmation of past research are supported. The analytic code for the current analyses is available at https://osf.io/5v9wu/?view_only=b45da1a33def47398e841a8966b0e0d0 Requests to access these datasets should be directed to Kayla Mansour, kayla.mansour@deakin.edu.au.

## Ethics Statement

The studies involving human participants were reviewed and approved by Deakin University, Faculty of Health, Human Research Ethics Advisory Group. The patients/participants provided their written informed consent to participate in this study.

## Author Contributions

KM, JM, and CG: study conceptualization and design. JM and CO: longitudinal MAPP study conceptualization and design. KM, JM, LF, and CG: data collection and preparation. KM, CG, and JM: study statistical analyses and interpretation of results. KM: writing of original draft. All authors critically reviewed, edited, and approved the final manuscript.

## Funding

KM undertook this research with support from a Deakin University Postgraduate Research (DUPR) Scholarship. CO was supported by an NHMRC Investigator Grant (APP1175086). JM was supported by a Deakin University Mid-career Fellowship.

## Conflict of Interest

The authors declare that the research was conducted in the absence of any commercial or financial relationships that could be construed as a potential conflict of interest.

## Publisher's Note

All claims expressed in this article are solely those of the authors and do not necessarily represent those of their affiliated organizations, or those of the publisher, the editors and the reviewers. Any product that may be evaluated in this article, or claim that may be made by its manufacturer, is not guaranteed or endorsed by the publisher.
