# Supplementary material for: Pre-pandemic Predictors of Loneliness in Adult Men During COVID-19
Source: Front Psychiatry. 2021 Dec 8;12:775588. doi: 10.3389/fpsyt.2021.775588 (PMC8692260; doi:10.3389/fpsyt.2021.775588)
Supplement: Supplementary file 2 [file Table_2.docx]

| *Supp Table 2. Moderation by state of residence* | | |
| --- | --- | --- |
| **Variable** | **Interaction** | |
|  | **Coefficient** | ***p*** |
| Depression | -0.10 | 0.372 |
| Anxiety | -0.04 | 0.740 |
| Stress | -0.05 | 0.675 |
| Irritability | -0.02 | 0.861 |
| State Anger | -0.02 | 0.861 |
| Career Orientated Identity Salience | 0.22 | 0.057 |
| Extraversion | -0.01 | 0.941 |
| Agreeableness | -0.15 | 0.219 |
| Conscientiousness | 0.03 | 0.805 |
| Neuroticism | -0.01 | 0.894 |
| Openness | 0.17 | 0.169 |
| Honesty/Humility | -0.03 | 0.781 |
| Trait Anger | 0.13 | 0.270 |
| Socially Prescribed Perfectionism | -0.08 | 0.475 |
| Environmental Mastery | -0.01 | 0.625 |
| Purpose in Life | -0.05 | 0.637 |
| Overall Physical Health | -0.04 | 0.754 |
| Job Competence | -0.02 | 0.475 |
| Home Competence | 0.06 | 0.524 |
| Hours Spent with Friends | 0.02 | 0.874 |
| Overall Social Support | 0.07 | 0.496 |
| Father Care | -0.13 | 0.266 |
| Father Control | 0.16 | 0.185 |
| Mother Care | 0.09 | 0.450 |
| Mother Control | -0.05 | 0.673 |
